# Two New Species of *Lactifluus* (Basidiomycota, Russulales) Sections *Piperati* and *Albati* Inhabiting Tropical *Quercus* Forests of Eastern Mexico

**DOI:** 10.3390/jof12030203

**Published:** 2026-03-11

**Authors:** Victor M. Bandala, Paloma Susan, Antero Ramos, Leticia Montoya

**Affiliations:** Red Biodiversidad y Sistemática, Instituto de Ecología, A.C., Carretera Antigua a Coatepec 351, El Haya, Xalapa 91073, Veracruz, Mexico; victor.bandala@inecol.mx (V.M.B.); paloma.susan@inecol.mx (P.S.); antero.ramos@inecol.mx (A.R.)

**Keywords:** neotropical fungi, milkcaps, tropical fungi, ectomycorrhizal fungi, taxonomy

## Abstract

Mexican species of *Lactifluus* have often been identified using names of morphologically similar Old World taxa. However, integrative approaches combining morphological and molecular data have revealed a high level of previously unrecognized diversity in the region. Here, two new species from lowland tropical *Quercus* forest are described: one in section *Piperati* (subgenus *Lactifluus*), characterized by pale yellow basidiomes and another in section *Albati* (subgenus *Lactariopsis*), with whitish basidiomes. The two taxa are distinguished by a unique set of macro- and micromorphological features, and their recognition is strongly supported by phylogenetic evidence from a concatenated dataset including nc ITS rDNA, nc 28S rDNA and the 6–7 region of the second largest subunit of the RNA polymerase II (*rpb2*).

## 1. Introduction

*Quercus* species are globally recognized for their exceptional diversity, and Mexico represents one of the two recognized centers of diversification of the genus [1], where these species are adapted to a wide range of environmental gradients, including lowland tropical areas. In the coastal plain of Veracruz state, eastern Mexico, tropical *Quercus* forests (TQFs) exist from altitudes close to the sea level to about 1000 m elevation. This ecosystem is peculiar for integrating both temperate and tropical biodiversity. Unfortunately, in Central Veracruz, primary vegetation, such as TQFs, has been drastically reduced to small, isolated remnants. This extensive loss and fragmentation have had profound consequences for landscape structure, ecosystem services, and regional biodiversity.

Among the key biotic components of TQF remnants, ectomycorrhizal (ECM) fungi play a fundamental role in maintaining ecosystem functioning. Systematic monitoring conducted by our research group in Veracruz has led to the discovery and description of new ECM species, including milkcaps and taxa of *Cantharellus*, *Phylloporus*, and *Tylopilus*, highlighting the remarkable diversity of ECM fungi, including endemic lineages. Given the ecological importance and increasing vulnerability of TQF, documenting and conserving their biological diversity is important.

One of the ECM fungal groups found in TQFs is the milkcap genus *Lactifluus* (Pers.) Roussel, a predominantly tropical lineage that also extends into temperate regions. *Lactifluus* is now recognized as a highly diverse genus, comprising several species complexes [2,3], reflecting extensive morphological convergence and hidden phylogenetic diversity. Among the most important are the *L. piperatus* complex (section *Piperati*) and the *L. vellereus* complex (section *Albati*), two speciose lineages that include several morphologically similar but phylogenetically distinct taxa. These groups have historically been treated under broad morphological species concepts, and their names have frequently been applied to collections from outside Europe, thereby obscuring the diversity of the genus in tropical regions, including Mexico. Recent studies have highlighted the genus diversity in South America [2,4,5,6,7,8,9], Central America and the Caribbean [3,10]. In Mexico, *L. lorenae* Montoya, Caro, Ramos & Bandala and *L. mexicanus* Montoya, Caro, Bandala & Ramos were described within the subgenus *Lactifluus*, the former in section *Piperati* and the latter in sect. *Lactifluus*. Both species were discovered in a TQF of central Veracruz state in eastern Mexico [11].

In the present study, we describe two additional species of *Lactifluus* from the TQF: one belonging to section *Piperati* (subgenus *Lactifluus*) and the other to section *Albati* (subgenus *Lactariopsis*), both occurring in the same area. Species delimitation was based on both morphological characters and molecular phylogenetic analyses. Their discovery enriches the known mycobiota of the region and highlights the ecological distinctiveness and conservation importance of TQFs as reservoirs of endemic and understudied fungal taxa.

## 2. Materials and Methods

### 2.1. Sampling

During the rainy season, from June to October of 2024–2025, field surveys were conducted in a relict tropical *Quercus* forest in central Veracruz (eastern Mexico), in the municipalities of Zentla (837–850 m.a.s.l.) and Alto Lucero (400–500 m.a.s.l.). At both privately owned sites, *Q. oleoides* Schltdl. & Cham. dominates the canopy, forming monodominant stands. At the Zentla site, *Quercus glaucescens* Bonpl. and *Q. sapotifolia* Liebm. are also present in monodominant stands.

### 2.2. Morphological Study

Macro-morphological features and colors were analyzed and recorded from fresh samples in different growth stages. Alpha-numeric color codes in descriptions follow Kornerup and Wanscher [12] (e.g., 7C8) and Munsell [13] (e.g., 10YR 8/6). Basidiomes were dried with a hot air dehydrator (45 °C) over the span of a week. Measurements and colors of micromorphological structures were recorded in 3% potassium hydroxide (KOH) and Melzer’s solution. The basidiospores were analyzed under a scanning electron microscope (SEM) FEI, Quanta 250 FEG (Hillsboro, OR, USA), following Montoya and Bandala [14]. Methods to determine basidiospore ranges are those used by Montoya et al. [15]. Thirty-five basidiospores per collection were measured (length and width of the spore in lateral view, excluding the ornamentation). These measurements are presented in taxonomic descriptions accompanied by the symbols: $\bar{X}$ representing the range of X (where X is the average of basidiospore length and width in each collection) and $\bar{Q}$ refers to the range of Q values (where Q represents the mean length/width ratio of basidiospores calculated for each collection). Line drawings were made with the aid of a drawing tube. Collections are part of the herbarium of the Instituto de Ecología, A.C., Xalapa, Mexico (XAL) [16].

### 2.3. Molecular Analysis

Genomic DNA was extracted from dried basidiome tissue, using the Exgene Plant SV mini extraction kit (GeneAll Biotechnology, Co., Ltd.; Seoul, Republic of Korea) according to Ramos et al. [17]. Polymerase chain reaction (PCR) was performed to amplify the internal transcribed spacer region of ribosomal DNA (ITS) using primers ITS1F-ITS5/ITS4 [18,19], the ribosomal large subunit 28S region (LSU), using primers LR0R/LR7 [20], and the region between the conserved domains 6 and 7 that encode the second largest subunit of RNA polymerase II (*rpb2*) was amplified with primers bRPB2 6f/fRPB2 7CR [21,22]. PCR conditions followed those in Montoya et al. [11] and Lebel et al. [23]. The PCR products were sent for sequencing by capillary electrophoresis to Macrogen, Inc. (Seoul, Republic of Korea). The obtained sequences were assembled, edited, and deposited in the GenBank database [24].

### 2.4. Phylogenetic Analysis

Preliminary analyses placed the new species within *Lactifluus* subgenus *Lactifluus* and subgenus *Lactariopsis*. Phylogenetic analyses were performed with the newly generated sequences and the sequences retrieved from GenBank [24], derived from the BLAST (https://blast.ncbi.nlm.nih.gov/Blast.cgi, accessed on 15 January 2026) search (best match) of related *Lactifluus* species, complemented with other GenBank sequences of species of all the sections within *Lactifluus* subgenus *Lactifluus* and subgenus *Lactariopsis* considered by De Crop et al. [2], Delgat et al. [10] and Lebel et al. [25]. We built a concatenated dataset of ITS, 28S and *rpb2* sequences, using PhyDE v.0.9971 [26], each individually aligned with MAFFT [27] and corrected inconsistencies manually to avoid any bias during the manipulation of the alignment. These alignments were concatenated. The evolutionary model was calculated with the IQ-Tree 2.3.5 package [28,29,30], the best fit model selected using the Bayesian Information Criterion (BIC). We used this information to generate a phylogeny with the Maximum Likelihood (ML) method, Nearest Neighbor Interchange (NNI) heuristic, the TIM2+I+G4 evolutionary model, and supported by the results of Bootstrap analysis.

To confirm this phylogeny, another phylogenetic tree was generated by the Bayesian Inference (BI) method, with the MrBayes v. 3.2.7 program [31], using the previously mentioned evolutionary model. Both phylogenies were displayed using FigTree v1.4.4 [32]. Only bootstrap scores (BSs) of ≥70% and Bayesian posterior probabilities (BPPs) of ≥0.90 were considered and indicated on the tree branches (BS/BPP) of the ML phylogeny obtained.

## 3. Results

**Figure 1:**
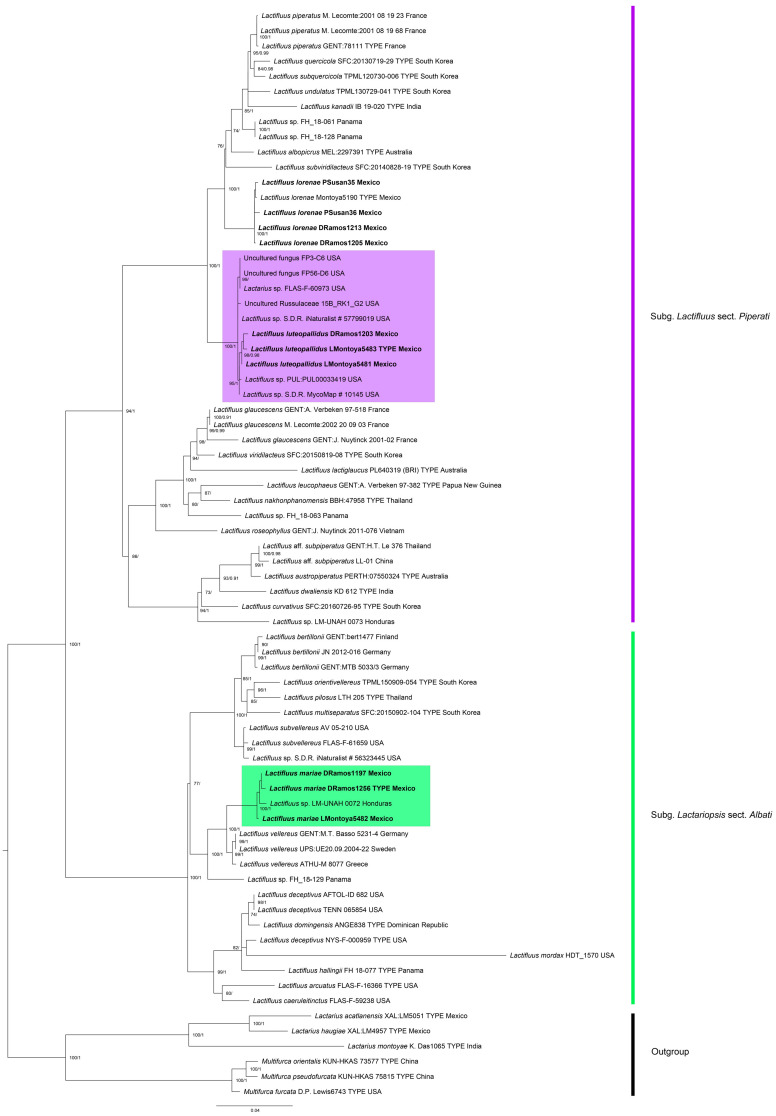
Concatenated three-locus (nc rDNA ITS, 28S and *rpb2*) phylogenetic analysis by maximum likelihood of *Lactifluus* species. Bootstrap scores (only values ≥ 70)/Posterior probabilities (only values ≥ 0.90) are indicated above branches. New species are indicated in bold: *Lactifluus luteopallidus *in the purple clade and *L. mariae* in the green clade.

The dataset included a total of 144 sequences (78 from type specimens) and 18 sequences from *Lactarius* and *Multifurca* species as the outgroup (Table 1). A total of 25 new sequences of *Lactifluus* species were generated in this study, nine of ITS, nine of the 28S region of rDNA and seven of *rpb2*. The molecular phylogeny (Figure 1) revealed that the produced sequences from Mexican specimens clustered in two strongly supported clades, with distinct phylogenetic positions in *Lactifluus*. Based on this evidence and on morphological characteristics, we propose the recognition of two new species, one in subgenus *Lactifluus* section *Piperati* and the other in subgenus *Lactariopsis* section *Albati*. Moreover, sequence analysis revealed the presence of four additional specimens of *L. lorenae*, a species previously described in section *Piperati*, from the TQF [11].

### Taxonomy

**Figure 2:**
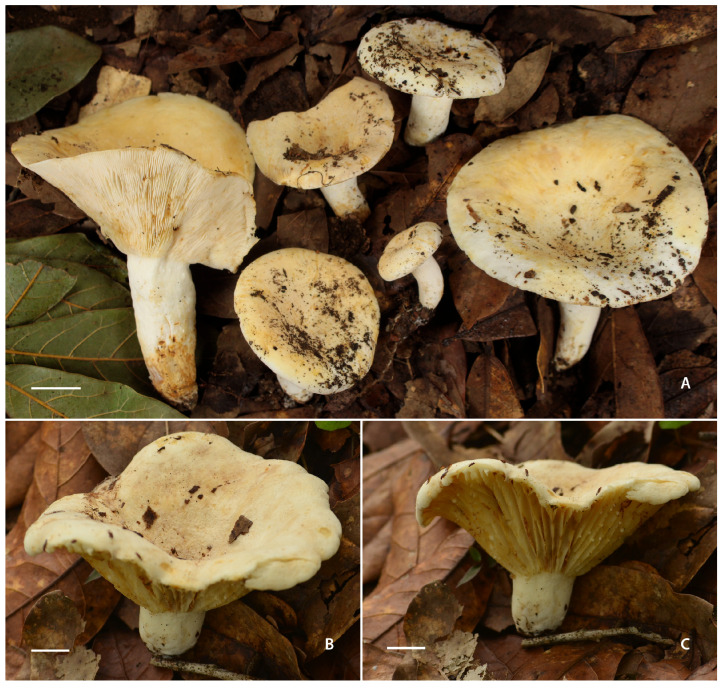
Basidiomes of *Lactifluus* species. (**A**) *Lactifluus luteopallidus* (holotype, Montoya 5483). (**B**,**C**) *Lactifluus mariae* (holotype, DRamos 1256). Scale bars: (**A**–**C**) 10 mm.

**Figure 3:**
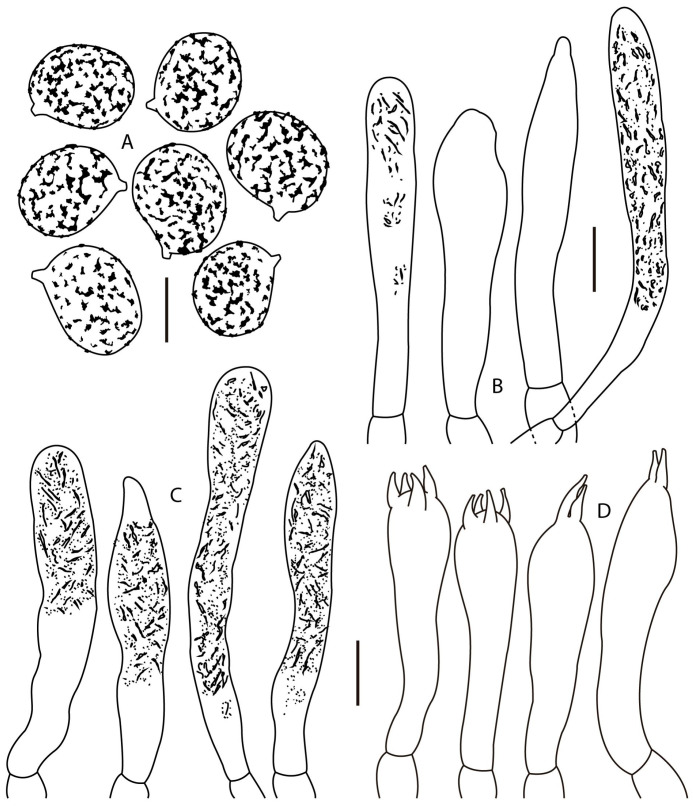
*Lactifluus luteopallidus* (holotype, Montoya 5483). (**A**) Basidiospores, (**B**) pleurocystidia, (**C**) cheilocystidia, and (**D**) basidia. Scale bars: (**A**) 5 μm, (**B**–**D**) 10 μm.

**Figure 4:**
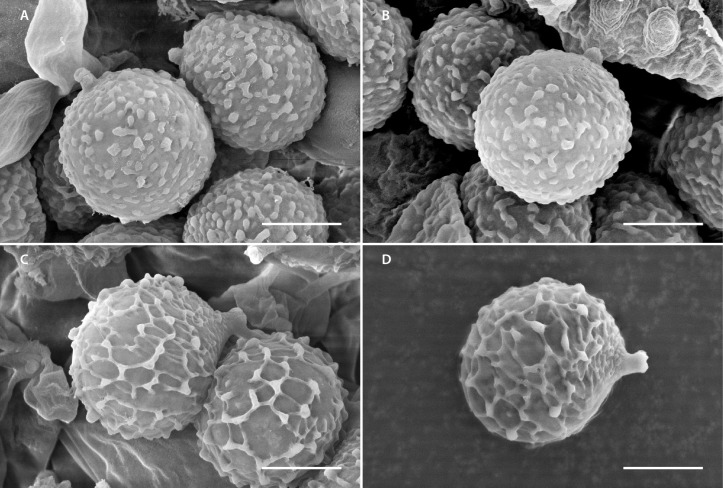
SEM microphotographs of *Lactifluus* species. (**A**,**B**) *Lactifluus luteopallidus* (holotype, Montoya 5483). (**C**,**D**) *Lactifluus mariae* (holotype, DRamos 1256). Scale bar (**A**–**D**) 3 µm.

**Figure 5:**
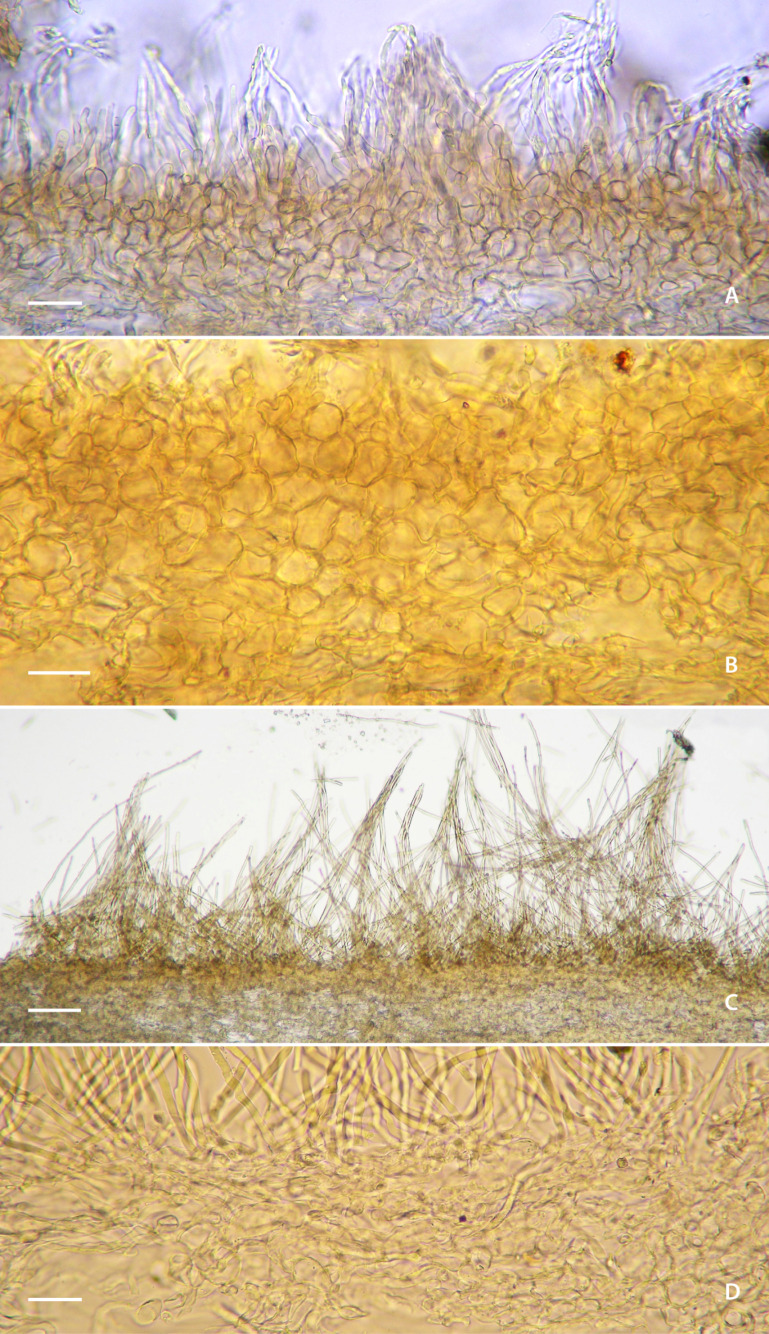
Pileipellis of *Lactifluus* species. (**A**,**B**) *Lactifluus luteopallidus* (holotype, Montoya 5483). (**C**,**D**) *Lactifluus mariae* (holotype, DRamos 1256). Scale bars: (**A**) 100 µm, (**B**) 10 µm, and (**C**,**D**) 25 μm.

*Lactifluus luteopallidus* Montoya & Bandala sp. nov. Figure 2A, Figure 3, Figure 4A,B and Figure 5A,B

MycoBank: 861976

Holotype. MEXICO, Veracruz State, Alto Lucero , 12 km SW Palma Sola (road Cardel-Nautla) 19 September 2025, Montoya 5483 (XAL). Ectomycorrhizal, under *Quercus sapotifolia*.

Diagnosis. Basidiomes pale yellow; lamellae adnate and crowded; unstaining context; odor like white glue or fishy; basidiospores 7–9(–9.5) × (5.5–)6–7(–7.5) µm, finely verrucose, ornamentation up to 0.5 µm high; cystidia versiform, with rounded apex or attenuated and even mucronate; pileipellis a palisade.

Etymology. In reference to the pale-yellow color of the pileus.

Pileus 23–70 mm diam., circular, plane depressed at center, infundibuliform when mature, surface dry pruinose, somewhat fibrillose, mostly smooth but somewhat rugose, faintly wrinkled towards central area, pale yellow (5Y8/3, 5Y8/1) over a whitish ground; margin incurved even when mature or straight when mature, edge entire or lobulate. Lamellae 5–15 mm broad, adnate, crowded, furcate at different levels of the hymenophore, thick, whitish with yellow (2.5Y8/2, 5Y8/3) tinges, with some ochraceous stains, with lamellulae of different sizes. Stipe 26–58 × 7–20 mm, attenuated towards the base, straight or somewhat bent, dry, finely pruinose, surface irregular, at times with faint longitudinal ribs towards the apex, whitish with yellow (5Y8/3) tinges. Context of pileus and stipe continuous, whitish, unstaining, only occasionally staining reddish green by oxidation at the stipe base. Latex white, milky, staining white paper yellow with a grayish hue after some hours. Odor like white glue or fishy; taste of context and latex burning acrid. KOH staining the pileus and stipe yellow to pale reddish, negative on context. NH_4_OH staining reddish the pileus and stipe surfaces, negative on context. Clamp connections absent.

Basidiospores 7–9(–9.5) × (5.5–)6–7(–7.5) µm, $\bar{X}$ = 7.4–8.4 × 6–6.6 µm, $\bar{Q}$ = 1.2–1.3 ellipsoid to broadly ellipsoid, finely verrucose, some with isolated short ridges, sometimes warty elements joined forming lines, but not forming a reticulum, ornamentation up to 0.5 µm high, suprahilar plage inamyloid, thin-walled. Basidia 42–63(–69) × 9–11 µm, clavate, tetrasporic or at times bisporic, hyaline, some with refractive content, thin-walled, unclamped. Pleuromacrocystidia 42–71(–77) × 7–12 µm, clavate to subcylindrical, apex rounded, frequently sinuous, at times mucronate or attenuated towards the apex, attenuated towards the base, thin-walled, translucid, frequently with refractive needle-like content, frequent or scarce. Cheilomacrocystidia 36–73 × 7–11 µm, subcylindrical to clavate, somewhat attenuated towards the apex, some broadly mucronate, like pleurocystidia, thin-walled, hyaline, frequently with abundant refractive needle-like content, frequent. Pseudocystidia absent. Pileipellis a palisade; suprapellis 50–112 µm thick, composed of anticlinally oriented hyphae, sometimes forming mounds, other hyphae intermixed; hyphae 44–123 × 2–5 µm, cylindrical, apex rounded or attenuated, straight or sinuous, septate, growing in chains, with a faint yellowish content, thin walled; subpellis a pseudoparenchymatous layer 60–100 µm thick, composed of subisodiametric or subellipsoid cells, 20–32 × 13–20 µm, hyaline to pale yellowish when seen in groups, wall 0.5–1 µm thick; dermatocystidia 35–65(–75) × 5–8 µm, at times present, hyaline with refractive needle-like and granular content, thin-walled, growing from subpellis cells, some of these latter with granular or needle like content. Context hyphae 4–8 µm broad, cylindrical, thin-walled, some hyphae with refractive granular or needle-like content, sphaerocytes 18–40 µm diam., hyaline, arranged in rosettes, more frequent towards the subpellis, laticiferous hyphae 7–17 µm broad, thin-walled or some with the wall up to 0.5–1 µm thick, with granulose dense content, some bifurcate. Hymenophoral trama irregular, composed of sphaerocytes 7–27 µm diam., hyaline, thin-walled; with intermingle hyphae 3–10 µm broad, hyaline, cylindrical, thin-walled, scarce; laticiferous hyphae 78–14 µm broad, with dense content, frequent. Clamp connections absent.

Habitat. Gregarious, under *Quercus sapotifolia* and *Q. oleoides*, infrequent.

Additional studied material. MEXICO, Veracruz, Alto Lucero , 12 km SW Palma Sola (road Veracruz-Nautla) 4 July 2024, Montoya 5481; 4 September 2024, DRamos 1203 (XAL).

*Lactifluus luteopallidus* belongs to section *Piperati* (subgenus *Lactifluus*). Despite the apparent morphological resemblance of this species and *L. lorenae* [11], the closest American taxon of the *L. piperatus* group, a detailed macro and micromorphological examination of basidiomes, revealed a distinct set of features that support their recognition. First, they differ in habit, particularly in color and in the hymenophore attachment. *Lactifluus luteopallidus* is pale yellow, whereas *L. lorenae* is white; the hymenophore is adnate in the former, compared to adnate to subdecurrent in the latter. Additionally, the context of the new species does not stain (only occasionally exhibiting a reddish green hue at the stipe base. In contrast, *L. lorenae* displays a brownish-orange staining when exposed. These species also differ in odor: *L. luteopallidus* emits a scent reminiscent of white glue or fish, while *L. lorenae* has a chlorine-like odor. Microscopic features further distinguish between the two species: the basidiospores of *L. luteopallidus* are mostly finely verrucose as seen under a light microscope and under SEM, whereas the basidiospores of *L. lorenae* exhibit a complete or incomplete reticulum. Other important distinguishing features include the morphology of the cystidia apex, which are rounded, attenuate, or even mucronate in *L. luteopallidus*, but are consistently rounded in *L. lorenae*. Finally, the pileipellis of *L. luteopallidus* is a palisade, whereas *L. lorenae* features a hyphoepithelium.

The phylogeny (Figure 1) inferred *Lactifluus luteopallidus* as sister to the Korean *L. subviridilacteus* H. Lee & Y.W. Lim [33], which differs in the basidiome habit (white pileus up to 120 mm diam and slightly decurrent lamellae), and especially in the latex that turns grayish green (white and staining white paper yellow in *L. luteopallidus*). The Korean species also has shorter basidiospores (up to 8.7 µm long), somewhat more globose (Q 1.11–1.24) and ornamented with an incomplete reticulum. Its pleuromacrocystidia are longer (113.5 µm), and the pileipellis is composed of a hyphoepithelium.

**Figure 6:**
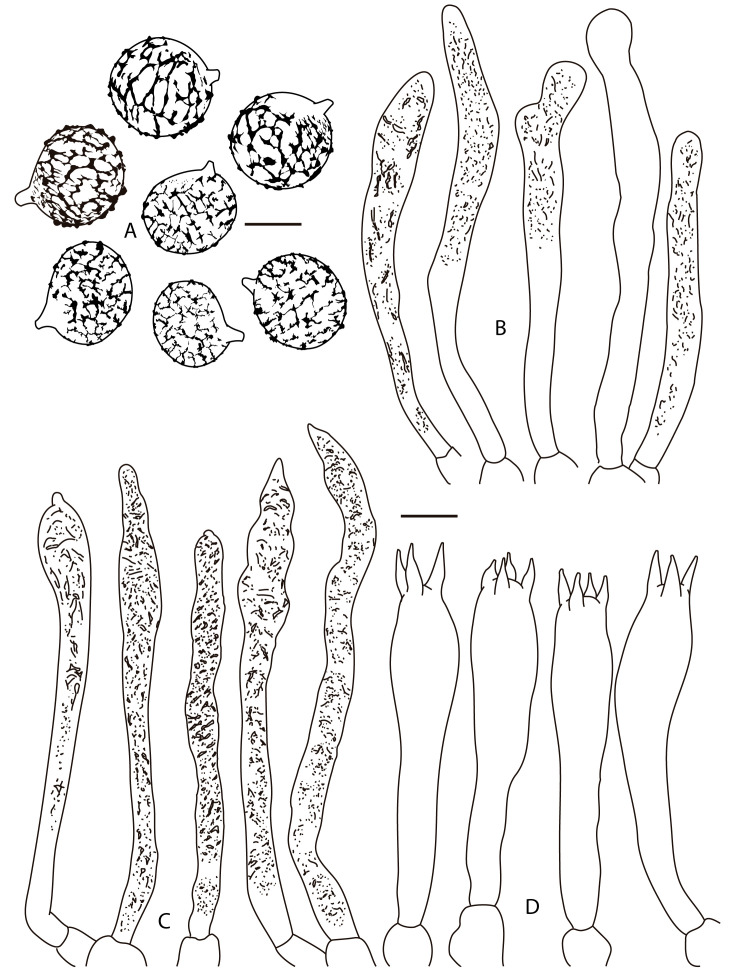
*Lactifluus mariae* (holotype, DRamos 1256). (**A**) Basidiospores, (**B**) pleurocystidia, (**C**) cheilocystidia, (**D**) basidia. Scale bars: (**A**) 5 μm, (**B**–**D**) 10 μm.

*Lactifluus mariae* Montoya & Bandala, sp. nov. Figure 2B,C, Figure 4C,D, Figure 5C,D and Figure 6

MycoBank: 861977

Holotype. MEXICO. Veracruz: Municipality of Zentla, near town of Zentla, 850 m.a.s.l., gregarious ectomycorrhizal, growing under *Quercus sapotifolia*, 23 September 2025, DRamos 1256 (XAL).

Diagnosis. Basidiomes whitish; pileus 71–100 mm diam, stipe 20–30 × 15–20 mm; lamellae adnate to subdecurrent, subdistant to distant; Latex white, unstaining, acrid to burning acrid; basidiospores (7–)7.5–9(–9.5) × (6–)6.5–8 µm, broadly ellipsoid, with a complete or incomplete amyloid reticulum, up to 1 µm high; cystidia subcylindrical to subclavate, sinuous, subfusoid, frequently mucronate at apex; pileipellis a lampropalisade, terminal elements attenuated towards the apex.

Etymology. Named after Mrs. Maria Cantón, an enthusiastic mushroom lover, who contributes to the conservation of TQF remnants and kindly shares her knowledge with us.

Pileus 71–100 mm diam, circular, at times reniform, plane, center depressed to infundibuliform, dry, hirsute, villous to subtomentose, velvety, firm, whitish (1A2, 3A1-2, 4A1-2; 2.5Y 6/3) with some dark (2.5Y 8/2, 8/8; 63-5D) areas, with reddish-ochraceous stains; margin decurved, continuous to irregularly finely lobulate. Lamellae subdistant to distant, adnate to subdecurrent, arcuate, 3–10 mm broad, thick, margin entire or faintly fimbriate, some bifurcate, cream to yellowish or yellowish-ochraceous (4A4) with age, with grayish-reddish to ochraceous stains, toward the zone close to the stipe with some interparietal connections or forked, with lamellulae of different lengths. Latex white, milky, not staining white paper, acrid to burning acrid. Stipe 20–30 × 15–20 mm, cylindrical, central or eccentric, attenuated towards the base, base abrupt, surface subvelvety, irregular, white (4A1-2), spotted grayish-ochre. Context of pileus and stipe continuous, whitish, unstaining. Odor of oil or chlorine; taste of context burning acrid or mild when no latex is present. KOH faintly orange on pileus, pale orange (5A3) on lamellae, yellow (8A7) or faintly orange on stipe.

Basidiospores (7–)7.5–9(–9.5) × (6–)6.5–8 µm,$\;\bar{X}$ = 8.1–8.31× 6.8–7.2 µm, $\bar{Q}$ = 1.16–1.18, broadly ellipsoid, thin-walled, with a complete reticulum, up to 1 µm high, with a fine mesh and warts or irregular bulges, suprahilar plage inamyloid. Basidia 55–82(–89) × (7–)9–13(–14) µm, clavate, attenuated towards the base, tri and tetrasporic, thin-walled, translucid, some with thin granular and needle-like refractive content, thin-walled. Pleuromacrocystidia 56–111(–118) × 5–10 µm, subcylindrical to subclavate, sinuous, subfusoid, frequently mucronate at apex, faintly yellowish, with refractive granular and needle-like content, thin-walled, abundant. Cheilomacrocystidia 35–74(–80) × 5–8(–9) µm, frequent, subclavate to subcylindrical, some sinuous, with rounded apex, others subfusoid, at times faintly mucronate, faintly yellowish, with dense and refractive granular and needle-like content, thin-walled. Pileipellis a lamprotrichoderm, terminal elements up to 220–550 × 4–6 µm, 4–10 µm broad towards the base, mostly cylindrical and attenuated towards the apex, at times septate, translucid with a faint yellowish green tint, wall 1–2 µm thick, slightly thinner towards the apex; growing from cylindrical or ventricose or irregularly inflated cells, up to 25 µm broad, thin-walled, these latter connected to hyphae 4–10 µm broad, cylindrical or at times bifurcate, some inflated to subisodiametric cells present, which are 14–20 µm diam., thin walled or wall up to 1 µm thick. Context composed of cylindrical hyphae, 4–10 µm broad, thin-walled, translucid, some cells with refractive content; sphaerocytes 14–25(–32) µm diam, frequent, hyaline, thin-walled or faintly thickened 0.5–1 µm, laticiferous hyphae 6–12 µm broad. Hymenophoral trama irregular, composed of sphaerocytes 22–32 × 14–25, with thin or thick wall 0.5–1 µm thick, hyaline; with inflated hyphae 7–10 µm diam, less frequent 4–6 µm diam, with wall up to 0.5 µm thick; with frequent laticiferous hyphae 6–12 µm broad, pale yellowish, with refractive content, thin walled, scarce. Clamp connections absent.

Habitat. Gregarious, under *Quercus oleoides* and *Q. sapotifolia*, infrequent.

Additional studied material. MEXICO. Veracruz: Municipality of Zentla, around town of Zentla, 850 m.a.s.l., 15 September 2024, Montoya 5482; 6 July 2024, DRamos 1197 (XAL).

*Lactifluus mariae* is a neotropical species, sister to the European *L. vellereus* (Fr.) Kuntze in section *Albati* (subgenus *Lactariopsis*). The taxonomy and nomenclature of the latter species have been progressively re-evaluated. Based on distribution and detailed morphological revision, Kytovuori and Korhonen [34] recognized *L. vellereus* sensu stricto as a species with large, robust, hirsute and white (to ochraceous in the pileus center) basidiomes. They emphasized the diagnostic value of its “mild latex (when separate from the flesh), long and thick hairs on the pileus and stipe and large, subglobose, ±reticulate spores”. These latter characters were used by Kytovuori and Korhonen [34] when distinguishing *L. vellereus* from the related *L. bertilloni* [as *Lactarius bertillonii* (Neuh. ex Z. Schaefer) M. Bon], in Sweden (where *L. vellereus* was described by Fries) and other regions of Fennoscandia.

When comparing *Lactifluus mariae* with *L. vellereus*, based on the information provided by Kytovuori and Korhonen [34], the Mexican species differs markedly in morphological and ecological features. Notably, *L. mariae* exhibits smaller basidiomes, with pileus reaching up to 100 mm diam. and stipe measuring up to 30 × 20 mm, in contrast to the larger basidiomes of *L. vellereus*, with the pileus reaching up to 300(–400) mm diam. and a stipe of 70 × 50 mm. Additionally, the latex of *L. mariae* is distinctly burning acrid. Microscopically, *L. mariae* produces shorter and narrower basidiospores, up to 9(–9.5) µm in length vs. 11.0 µm; and up to 8 µm in width vs. 9.3 µm. The basidiospores of *L. mariae* are also more globose, with a $\bar{Q}$ value of up to 1.18 vs. 1.24, and possess a more prominent reticulum, up to 1 µm in height vs. 0.2 µm in *L. vellereus*. Furthermore, the basidia of *L. mariae* are broader, reaching up to 13(–14) µm, whereas up to 11.5 µm in *L. vellereus*. Additional distinctions are supported by the description of *L. vellereus* provided by Heilmann-Clausen et al. [35] , who noted lamellae as “…medium crowded… when old or bruised turning clay-buff to dark brick coloured”; basidiospores measuring “7.7–12.0 × 6.3–9.6 μm… Q = 1.00–1.40, av. 1.12–1.28…” and pileipellis terminal elements “with a slightly inflated apex”. In contrast, *L*. *mariae* displays more distant lamellae, shorter and more globose basidiospores, and pileipellis elements that are attenuated at the apex. Ecologically, *L. vellereus* is associated with *Fagus*, *Quercus*, *Betula* and conifers in temperate, even hemiboreal or southern boreal regions [34,35].

## 4. Discussion

Earlier taxonomic studies on *Lactifluus* species belonging to sections *Albati* and *Piperati* focused primarily on European taxa, such as *L. piperatus* and *L. vellereus*, which for a long time were considered easily recognizable species and were even reported from other continents [36,37,38]. Other reports included cases related to poisoning incidents involving *L. vellereus* in China [39], as well as studies referring to the chemical composition of *L. piperatus* from Japan [40]. However, as morphological studies advanced, such as that by Kytovuori and Korhonen [34], and especially, with the integration of molecular phylogenetic analyses, cryptic diversity began to become apparent [3,41]. Subsequently, *Lactifluus piperatus* and *L. vellereus* were each recognized as species complexes that are difficult to recognize morphologically.

The phylogeny here obtained (Figure 1), in addition to supporting the distinction of the two species we have described, also revealed that *Lactifluus luteopallidus* clustered with seven unidentified sequences of *Lactifluus* from Indiana and Florida, USA. An additional sequence, identified as “*Lactarius* sp.” from Honduras [42], clustered with *L. mariae*, providing evidence for the occurrence of *L. mariae* in that country.

The distinction of the current species described and *L. lorenae* supports the hypothesis that there is no overlap in *Lactifluus* species diversity between continents [10]. *Lactifluus luteopallidus* appears as a sister lineage (with strong support) to a group of species related to the European *L. piperatus*, which includes the Mexican *L. lorenae* and other species from Australia, Central America and Asia. The second species, *L. mariae*, also forms a strongly supported clade, sister to the European *L. vellereus* (Fr.) Kuntze. Indeed, Delgat et al. [10] also observed that Central American clades of *Lactifluus* associated with Betulaceae, Fagaceae or Pinaceae have their closest relatives in North America, Asia and/or Europe. This is also supported by species occurring at very low elevations, such as those described here and those previously reported from TQF [11], such as *L. mexicanus*, which is close to the Asian *L. dissitus* [43]. The current diversity of ectomycorrhizal fungi and their host species in the TQF is a result of sustained evolutionary processes related to the paleoenvironmental dynamics in this Neotropical region. This is particularly the case for ectomycorrhizal host species of Holarctic origin, such as certain lineages of *Quercus* that have persisted in this tropical environment at elevations below 500 m close to the coast.

*Lactifluus lorenae* and *L. mexicanus* (section *Lactifluus*) were previously discovered in the TQF together with the two species described here, which constitute the first reports of *Lactifluus* in this ecosystem. The findings of these species in TQF remnants provide information on the ectomycorrhizal association of members of this fungal genus in tropical lowland conditions. Within this ecosystem, *L. lorenae* and *L. mexicanus* grow under *Q. oleoides*, while *L. luteopallidus* and *L. mariae* have been found under both *Q. oleoides* and *Q. sapotifolia*. Although their ectomycorrhizal status has not yet been confirmed through root tip analyses, the consistent occurrences of basidiomes in monodominant stands of each *Quercus* species strongly support the hypothesis that these fungi form ectomycorrhizal associations with such hosts.

## Figures and Tables

**Table 1:** *Lactifluus* taxa included in this study: voucher, isolates, strains, location and ITS, 28S, *rpb2* GenBank accession number. New sequences in bold.

Taxon	Voucher/Isolate/Strain	ITS	28S	*rpb*2	Locality	TYPE
**subg. ** ***Lactifluus*** ** sect. ** ***Piperati***						
*Lactifluus* aff. *subpiperatus*	GENT:H.T. Le 376	KF220110	-	-	Thailand	
*Lactifluus* aff. *subpiperatus*	LL-01	MT571520	-	-	China	
*Lactifluus albopicrus*	MEL:2297391	NR_171289	NG_073769	-	Australia	TYPE
*Lactifluus austropiperatus*	PERTH:07550324	NR_171290	NG_073776	-	Australia	TYPE
*Lactifluus curvativus*	SFC:20160726-95	NR_182695	NG_153884	MN215345	Republic of Korea	TYPE
*Lactifluus dwaliensis*	KD 612	KR364042	-	-	India	TYPE
*Lactifluus glaucescens*	GENT:A. Verbeken 97-518	KF220026	KF220129	-	France	
*Lactifluus glaucescens*	GENT:J. Nuytinck 2001-02	KF220022	-	-	France	
*Lactifluus glaucescens*	M. Lecomte:2002 20 09 03	KF220031	KF220134	KF220224	France	
*Lactifluus kanadii*	IB 19-020	MW295837	MW295839	MW354672	India	TYPE
*Lactifluus lactiglaucus*	PL640319 (BRI)	MW007414	-	-	Australia	TYPE
*Lactifluus leucophaeus*	GENT:A. Verbeken 97-382	GU258299	GU265640	GU258379	Papua New Guinea	TYPE
***Lactifluus lorenae***	**DRamos 1205**	**PX852611**	**-**	**PX851911**	**Mexico**	
***Lactifluus lorenae***	**LMontoya5328**	**PX852612**	**PX856289**	**PX851912**	**Mexico**	
***Lactifluus lorenae***	**PSusan 35**	**PX852613**	**PX856290**	**PX851913**	**Mexico**	
***Lactifluus lorenae***	**PSusan 36**	**PX852614**	**PX856291**	**-**	**Mexico**	
*Lactifluus lorenae*	Montoya5190	NR_173809	NG_068864	MK258872	Mexico	TYPE
***Lactifluus luteopallidus***	**DRamos1203**	**PX852608**	**PX856286**	**PX851910**	**Mexico**	
***Lactifluus luteopallidus***	**LMontoya5481**	**PX852609**	**PX856287**	**-**	**Mexico**	
***Lactifluus luteopallidus***	**LMontoya5483**	**PX852610**	**PX856288**	**PX851909**	**Mexico**	**TYPE**
*Lactifluus nakhonphanomensis*	BBH:47958	-	NG_229031	OP432870	Thailand	TYPE
*Lactifluus piperatus*	GENT:78111	KF220122	KF220215	-	France	TYPE
*Lactifluus piperatus*	M. Lecomte:2001 08 19 23	KF220120	KF220212	KF220285	France	
*Lactifluus piperatus*	M. Lecomte:2001 08 19 68	KF220119	KF241840	KR364453	France	
*Lactifluus quercicola*	SFC:20130719-29	MN215390	MN215346	MN212838	Republic of Korea	TYPE
*Lactifluus roseophyllus*	GENT:J. Nuytinck 2011-076	KF220107	KF220202	KF220276	Vietnam	
*Lactifluus* sp.	FH_18-061	MN102688	MN101705	MN120456	Panama	
*Lactifluus* sp.	FH_18-063	MN102690	MN101707	MN120458	Panama	
*Lactifluus* sp.	FH_18-128	MN102695	MN101712	MN120457	Panama	
*Lactifluus* sp.	FLAS-F-60973	MH016922	-	-	USA	
*Lactifluus* sp.	LM-UNAH 0073	HM639278	-	-	Honduras	
*Lactifluus* sp.	PUL:PUL00033419	OM473452	-	-	USA	
*Lactifluus* sp.	S.D. Russell iNaturalist # 57799019	OM473932	-	-	USA	
*Lactifluus* sp.	S.D. Russell MycoMap # 10145	OM473451	-	-	USA	
*Lactifluus subquercicola*	TPML120730-006	MN215395	MN215351	MN212843	Republic of Korea	TYPE
*Lactifluus subviridilacteus*	SFC:20140828-19	MN215397	MN215353	MN212845	Republic of Korea	TYPE
*Lactifluus undulatus*	TPML130729-041	MN215399	MN215355	MN212847	Republic of Korea	TYPE
*Lactifluus viridilacteus*	SFC:20150819-08	NR_182696	MN215358	MN212850	Republic of Korea	TYPE
Uncultured fungus	FP3-C6	MF946116	-	-	USA	
Uncultured fungus	FP56-D6	MF946237	-	-	USA	
Uncultured Russulaceae	15B_RK1_G2	KX899073	-	-	USA	
**subg. ** ***Lactariopsis*** ** sect. ** ***Albati***						
*Lactifluus arcuatus*	FLAS-F-16366	MK931344	-	-	USA	TYPE
*Lactifluus bertillonii*	GENT:bert1477	MH125229	-	-	Finland	
*Lactifluus bertillonii*	GENT:MTB 5033/3	MH125230	-	-	Germany	
*Lactifluus bertillonii*	JN 2012-016	KR364087	KR364217	KR364261	Germany	
*Lactifluus caeruleitinctus*	FLAS-F-59238	MK931345	-	-	USA	
*Lactifluus deceptivus*	AFTOL-ID 682	AY854089	AY631899	AY803749	USA	
*Lactifluus deceptivus*	NYS-F-000959	MN251093	-	-	USA	TYPE
*Lactifluus deceptivus*	TENN 065854	KR364101	-	KR364271	USA	
*Lactifluus domingensis*	ANGE838	MK931341	MN128990	MK937132	Dominican Republic	TYPE
*Lactifluus hallingii*	FH 18-077	MK931338	MN128991	MK937129	Panama	TYPE
***Lactifluus mariae***	**DRamos1197**	**PX852615**	**PX856293**	**PX851914**	**Mexico**	
***Lactifluus mariae***	**DRamos1256**	**PX852616**	**PX856294**	**-**	**Mexico**	**TYPE**
***Lactifluus mariae***	**LMontoya5482**	**-**	**PX856295**	**PX851915**	**Mexico**	
*Lactifluus mordax*	HDT 1570	MN251096	-	-	USA	TYPE
*Lactifluus multiseparatus*	SFC:20150902-104	MN215366	MN215317	MN212809	Republic of Korea	TYPE
*Lactifluus orientivellereus*	TPML150909-054	MN215369	MN215320	MN212812	Republic of Korea	TYPE
*Lactifluus pilosus*	LTH 205	KR364006	KR364134	KR364323	Thailand	TYPE
*Lactifluus* sp.	FH_18-129	MN102696	MN101713	MN120455	Panama	
*Lactifluus* sp.	LM-UNAH 0072	HM639276-HM639277	-	-	Honduras	
*Lactifluus* sp.	S.D. Russell iNaturalist #56323445	ON059206	-	-	USA	
*Lactifluus subvellereus*	AV 05-210	KR364010	KR364138	KR364347	USA	
*Lactifluus subvellereus*	FLAS-F-61659	MH212034	-	-	USA	
*Lactifluus vellereus*	ATHU-M 8077	KR364106	KR364237	KR364354	Greece	
*Lactifluus vellereus*	GENT:M.T. Basso 5231-4	KF220123	KF220216	KF220288	Germany	
*Lactifluus vellereus*	UPS:UE20.09.2004-22	DQ422034	DQ422034	DQ421936	Sweden	
**Outgroup**						
*Lactarius acatlanensis*	XAL:LM5051	NR_152976	NG_060332	KT736508	Mexico	TYPE
*Lactarius haugiae*	XAL:LM4957	NR_152975	NG_060331	KT736505	Mexico	TYPE
*Lactarius montoyae*	K. Das1065	EF560673	NG_060260	GU258380	India	TYPE
*Multifurca furcata*	D.P. Lewis 6743	MH063862	MH063830	MH061159	USA	TYPE
*Multifurca orientalis*	KUN-HKAS 73577	MH063856	MH063825	MH061154	China	TYPE
*Multifurca pseudofurcata*	KUN-HKAS 75815	MH063849	MH063819	MH061148	China	TYPE

## Data Availability

The original contributions presented in the study are included in the article, further inquiries can be directed to the corresponding author.

## Abbreviations

The following abbreviations are used in this manuscript:

TQF	Tropical *Quercus* Forest
RTP	Priority Terrestrial Region
ECM	Ectomycorrhizal
m.a.s.l.	meters above sea level
KOH	Potassium hydroxide
NH_4_OH	Ammonium hydroxide
SEM	Scanning Electron Microscope
XAL	Herbarium of the Instituto de Ecología, A.C., Xalapa, Mexico
DNA	Deoxyribonucleic Acid
PCR	Polymerase Chain Reaction
ITS	Internal Transcribed Spacer region of ribosomal DNA
LSU	Ribosomal large subunit 28S region
*rpb2*	Second largest subunit of RNA polymerase II
BIC	Bayesian Information Criterion
AIC	Akaike Information Criterion
ML	Maximum Likelihood
NNI	Nearest Neighbor Interchange
BI	Bayesian inference
BPP	Bayesian posterior probabilities
rDNA	Ribosomal DNA
BS	Bootstrap score
